# Association Between Patatin‐Like Phospholipase Do‐Main‐Containing Protein‐3 Variant and Cardiovascular Disease Risk in People Living With HIV on Antiretroviral Therapy in Taiwan: A Cross‐Sectional Study

**DOI:** 10.1002/hsr2.71875

**Published:** 2026-02-22

**Authors:** Chia‐Hui Yu, Gwo‐Tarng Sheu, Chien‐Feng Li, Win‐Long Lu, Hao‐Jan Yang, Hung‐Chang Hung, Yuan‐Ti Lee

**Affiliations:** ^1^ School of Nursing, College of Medicine Chung Shan Medical University Hospital Taichung Taiwan; ^2^ Institute of Medicine Chung Shan Medical University Taichung Taiwan; ^3^ Department of Internal Medicine, Chung Shan Medical University Hospital Division of Infectious Diseases Taichung Taiwan; ^4^ Department of Family and Community Medicine Chung Shan Medical University Hospital Taichung Taiwan; ^5^ Department of Public Health Chung Shan Medical University Taichung Taiwan; ^6^ Department of Internal Medicine, Division of Gastroenterology, Nantou Hospital Ministry of Health and Welfare Nantou Taiwan; ^7^ Department of Healthcare Administration Central Taiwan University of Science and Technology Taichung Taiwan; ^8^ School of Medicine Chung Shan Medical University Taichung Taiwan

**Keywords:** antiretroviral therapy, apolipoprotein E, cardiovascular disease, HIV, patatin‐like phospholipase do‐main‐containing protein‐3 variant, pharmacogenetics, polymorphisms

## Abstract

**Background and Aims:**

Cardiovascular disease (CVD) risk is elevated among people living with HIV (PLWH), particularly those receiving antiretroviral therapy (ART). This study aimed to examine associations between single‐nucleotide polymorphisms (SNPs) in lipoprotein‐related genes and CVD risk among PLWH undergoing ART.

**Methods:**

Blood samples from 337 PLWH at Chung Shan Medical University Hospital were analyzed, including 238 individuals who switched ART and 99 who continued their regimen. Genotyping of four SNPs—namely, ATP binding cassette B1 (*ABCB1*; rs1045642), apolipoprotein E (*APOE*; rs429358 and rs7412), and patatin‐like phospholipase domain‐containing protein 3 (*PNPLA3*; rs738409) was performed using real‐time polymerase chain reaction and sequence‐based typing. CVD risk scores were calculated using the D:A:D model, with high risk defined as a 10‐year risk > 5%. Associations between SNPs and CVD risk scores were assessed using analysis of covariance, adjusting for demographic and clinical covariates.

**Results:**

The cohort was predominantly male 95.6% (322/337), with a mean age of 34.6 years. Metabolic abnormalities were common, and 16.0% (54/337) of participants on ART were classified as high‐risk for CVD. Among the SNPs analyzed, *PNPLA3* (rs738409) was significantly associated with higher D:A:D (R) 10‐year CVD risk scores (*p* = 0.03) and showed marginal associations with 5‐year risk scores (*p* = 0.05). The *APOE* (rs7412) T allele demonstrated borderline associations with increased CVD risk (*p* = 0.07–0.09). No significant associations were observed for *ABCB1* or *APOE* (rs429358). *PNPLA3* may influence triglyceride hydrolysis via adipose triglyceride lipase, contributing to fatty liver disease and elevating CVD risk.

**Conclusion:**

SNPs in *PNPLA3* and *APOE* may contribute to increased CVD risk among PLWH receiving ART. Incorporating genetic screening into clinical care could support personalized treatment strategies and improve long‐term CVD outcomes.

## 1 Introduction

The ongoing impact of HIV/AIDS continues to pose a significant global public health challenge. The World Health Organization (WHO) estimates that by the end of 2024, 40.8 million (37.0–45.6 million) people were living with HIV (PLWH) globally; in that year, there were 1.3 million (1.0–1.7 million) new infections and 630,000 (490,000–820,000) HIV‐related deaths [1]. There is no cure for HIV infection. However, the impact of antiretroviral therapy (ART) in controlling viral replication and reducing transmission risk. With effective treatment, PLWH and those at substantial risk can achieve improved health outcomes, leading long and productive lives comparable to the general population [2, 3]. As life expectancy increases, these populations are affected by the effects of gradual aging and environmental risk factors, which are also known to affect the general population [2]. The long‐term complications of this disease are multifactorial and can be related to the virus itself or to the adverse effects of ART [3]. These effects can contribute to the development of obesity, diabetes mellitus (DM), and ultimately cardiovascular disease (CVD) [4, 5, 6, 7].

Previous studies have shown that adults infected with HIV have a higher risk of CVD than the general population [4, 5]. AIDS patients who switch antiviral drugs also have an increased risk of CVD [8, 9] and metabolic disorders [10, 11, 12]. Multiple studies have shown a high correlation between metabolism and CVD [10, 13, 14], indicating that there may be underlying genetic factors common to both [15].

Studies exploring the association between genetic polymorphisms and metabolic syndrome (MetS) and its components found that the apolipoprotein E (*APOE*; rs429358 and rs7412) genotype was associated with body mass index (BMI kg/m²) [16]. Research showed a significant correlation between the *APOE*(rs7412) genotype and both BMI and waist circumference after controlling for age, smoking status, and blood lipid levels [17]. Analysis of the patatin‐like phospholipase domain‐containing protein 3 (*PNPLA3*; rs738409) genotype showed an association with MetS, patients carrying CG and GG alleles showed higher incidences of hypertension, lipid abnormalities, fatty liver, and MetS compared to those with the CC allele [15]. Another study found a significant correlation between the *PNPLA3*(rs738409) genotype and elevated TG levels [18].

While an increasing number of genetic studies focus on the relationship between genes and lipid metabolism [15, 17, 18, 19], there are relatively few studies that investigate the relationship between genes and CVD.

Those that did found that the ATP‐binding cassette B1 (*ABCB1*; rs1045642) genotype is associated with hypertension and chronic kidney disease [20, 21]. In addition, the *PNPLA3*(rs738409) genotype is associated with coronary artery disease and risk factors for heart metabolism and may be a factor in atherosclerosis [22]. Dysfunctional lipid metabolism is one of the risk factors for CVD, and studies on the relationship between *APOE* genes, lipids, and myocardial infarction show that the *APOE* (rs7412 and rs429358) genotypes are significantly correlated with lipid levels [19]. Depending on the combination of alleles, such as *APOE* (rs7412 and rs429358), individuals can have one of six common *APOE* genotypes (ε4ε4, ε4ε3, ε4ε2, ε3ε3, ε2ε3, and ε2ε2); thus, it is necessary to understand the association between specific *APOE* genotypes and the outcome of the disease. In fact, *APOE* alleles ε3ε4 and ε4ε4 are associated with an increased risk of developing hypercholesterolemia and coronary heart disease [17].

Despite growing evidence, few studies have examined single‐nucleotide polymorphisms (SNPs) associated with hypertriglyceridemia or MetS undergoing ART in Chinese HIV patients. Our objective was to assess the association between specific SNPs in lipoprotein‐related genes—namely, *ABCB1*, *APOE*, and *PNPLA3*—and CVD risk in PLWH undergoing ART in Taiwan.

## 2 Materials and Methods

### 2.1 Subjects

At study initiation, 500 HIV‐infected individuals were receiving ART at Chung Shan Medical University Hospital (CSMUH), Taichung, Taiwan. After applying eligibility criteria, 337 patients were enrolled in this prospective study. Participants were recruited between September 2018 and February 2021. The study was approved by the Institutional Review Board of the CSMUH (CSMUH No: CS15074), and all participants provided their informed written consent upon enrollment. The inclusion criteria consisted of age ≥ 20 years and PLWH (ICD9 042 or ICD10 B20) who received ART in the past 6 months at CSMUH. The exclusion criteria included patient refusal, age < 20 years, PLWH without ART or discontinuation of ART for more than 6 months, and incomplete data (including study dropout and loss to follow‐up). In total, 163 individuals were excluded based on these criteria. HIV diagnoses were confirmed in individuals who tested positive using both enzyme‐linked immunosorbent assay and Western blot testing for HIV. The following participant data were collected: specimen collection and testing for assessment of CVD risk, and collection of demographic data, HIV history, comorbidity data, and lipid profiles. The recorded demographic data included gender, age, height, body weight, systolic and diastolic blood pressure, BMI, family history, and smoking status. Additionally, clinical characteristics and risk factors were documented, including active smoking, hypertension, type 1 and type 2 DM, the concomitant use of antidiabetic, antihypertensive, and antihyperlipidemic medications for more than 6 months, a history of myocardial infarction, stroke, invasive coronary artery procedures (such as coronary artery bypass grafting, angioplasty, or carotid artery endarterectomy), mortality due to coronary heart disease, and hypercholesterolemia [7].

An active smoker was defined as an individual who currently smokes at least one pack of cigarettes per day. Hypertension was identified as a systolic and/or diastolic blood pressure exceeding 140/90 mmHg or the administration of antihypertensive treatment during the study period. Hypercholesterolemia was characterized by serum total cholesterol levels surpassing 200 mg/dL [7]. DM was defined as HbA1c ≥ 6.5% (mmol/mol) or having received any oral antidiabetic agents or insulin treatment during the study period. The family history of CVD was obtained by inquiring whether any immediate family members—specifically parents, siblings, or children—had experienced a fatal or non‐fatal myocardial infarction.

Clinical laboratory data obtained from blood samples after a 12‐h fast included fasting plasma glucose (≥ 6.1 mmol/L; 110 mg/dL), total cholesterol (< 200 mg/dL), low‐density lipoprotein cholesterol (LDL‐C; < 100 mg/dL), high‐density lipoprotein cholesterol (HDL‐C; < 40 mg/dL), and triglycerides (TG; < 150 mg/dL). We obtained data on CD4+T cell counts (cells/mm³) and viral load values of HIV RNA (copies/ml) from baseline through 6 monthly follow‐up visits. Plasma HIV RNA viral load was measured using the Cobas TaqMan HIV‐1 assay (Roche Diagnostics Systems Inc.), and CD4+ lymphocyte counts were determined by flow cytometry (Beckman Coulter Inc.). Clinical data were collected at the time of enrollment, and subsequent laboratory data were collected at 6‐month follow‐up visits.

### 2.2 Evaluation of ART

The potential association between ART and CVD development has been investigated in previous studies [8, 9]. For this analysis, we selected participants from the overall cohort who were receiving a ART regimen at the time of enrollment. All participants were prescribed at least three antiretroviral drugs. The regimen was defined by the International Antiviral Society–USA Panel guidelines as: (1) two nucleoside reverse transcriptase inhibitors (NRTI) in combination with at least one nonnucleoside reverse transcriptase inhibitor (NNRTI), (2) two NRTI in combination with at least one protease inhibitor (PI) containing ritonavir, (3) two NRTI in combination with at least one PI, (4) two NRTIs in combination with an integrase inhibitor (INSTI), and (5) a regimen of one PI containing ritonavir in combination with one NRTI (lamivudine; 3TC) [3].

The treating clinicians determined the choice of therapy and any modifications to the regimen over the course of the study. Adherence, adverse events, and regimen adjustments were systematically monitored at each follow‐up visit and assessed through chart reviews. In the present study, baseline and recent ART were categorized by drug class, and the probability of adverse events caused by those who were receiving ART that increase the risk of CVD was analyzed. Baseline and recent ART were defined by the participants receiving drugs within 180 days of the baseline enrollment date and the date closest to CVD diagnosis, death, or the last follow‐up date, respectively [23].

### 2.3 Assessment of CVD Risk

Participants were evaluated for CVD risk factors based on the criteria established by the Data Collection on Adverse Events of Anti‐HIV Drugs (D:A:D) study. These criteria were used to estimate both 5‐ and 10‐year CVD risk for all participants [23]. The risk factors used in the 5‐ and 10‐year risk scores of CVD of D:A:D (R) include age, sex, previous smoker, current smoker, diabetes (diagnosis or antidiabetic treatment), CD4+T cell counts (cells/mm³), family history of CVD, total cholesterol, HDL cholesterol, and systolic blood pressure. Cutoffs (≥ 5% vs. < 5%) for 5‐ and 10‐year risk scores follow D:A:D study thresholds for identifying elevated CVD risk. These thresholds have been validated in large HIV cohorts and are commonly used in clinical and epidemiological studies to stratify risk. Furthermore, the full D:A:D (F) CVD prediction algorithm is also used to estimate the risk of an individual developing CVD within the next 5 and 10 years. The required information for this model includes sex, age, smoking status, diabetes (diagnosis or antidiabetic treatment), family CVD history, systolic blood pressure, total cholesterol, HDL, CD4+T cell counts (cells/mm³), cumulative exposure to PI, cumulative exposure to NRTI, and current use of abacavir. Composite CVD outcomes include myocardial infarction, stroke, invasive coronary artery procedures (including coronary artery bypass or angioplasty and carotid artery endarterectomy), or death from coronary heart disease. The D:A:D (F) model is valid for 18 to 75‐year‐old PLWH, with cumulative exposure to NRTI up to approximately 8 to 10 years, and exposure to PI up to approximately 5 to 6 years. Extrapolating beyond these exposure times without recalibrating leads to an overestimation of CVD risk. The D:A:D (R) model is recommended for individuals who were highly exposed to ART [23].

### 2.4 Serum Collection and Storage

All blood samples were collected under sterile conditions and without anticoagulant. Serum samples were stored immediately after collection. Sera were collected and stored in a freezer at −80°C after centrifugation at 800 × g at 4°C for 10 min [24].

### 2.5 Blood Sample Collection and Genomic DNA Extraction

Blood samples were collected from PLWH who were receiving ART and stored in biospecimen banks at CSMUH. Genomic DNA was extracted from anticoagulated venous blood treated with ethylenediaminetetraacetic acid (EDTA) using the QIAamp DNA Blood Mini Kit (Qiagen, Valencia, CA, USA), following the manufacturer's protocol [24]. DNA was dissolved in Tris‐ethylene buffer (10 mmol/L Tris and 1 mmol/L EDTA; pH 7.8) and subsequently quantified using a spectrophotometer at an optical density of 260 nm (OD260). The final preparation was stored at −20°C for polymerase chain reaction (PCR) experiments [24].

### 2.6 Selection of Lipoprotein Gene Polymorphisms

Single nucleotide polymorphisms including the *APOE* alleles (ε4, ε3, and ε2), a combination of variants at two SNP sites (rs429358 and rs7412), *ABCB1* (rs1045642) gene, and *PNPLA3* (rs738409) gene were selected in this study as these SNPs were likely to affect lipid metabolism and CVD development [18, 25, 26, 27, 28].

### 2.7 Determination of SNPs in Biomarkers by Real‐Time PCR and Genotyping

Allelic discrimination of SNPs in biomarkers was performed using the ABI StepOne™ Real‐Time PCR System (Applied Biosystems, Foster City, CA, USA) and analyzed with SDS version 3.0 software (Applied Biosystems) through the TaqMan genotyping assay. Each PCR reaction consisted of 5 μL of TaqMan Genotyping Master Mix, 0.25 μL of TaqMan Probe Mix, and 10 ng of genomic DNA. The thermal cycling conditions included an initial denaturation step at 95°C for 10 min, followed by 40 amplification cycles of 95°C for 15 s and 60°C for 1 min [24]. A detailed description of the SNP detection methodology using real‐time PCR and genotyping has been previously documented in prior research [24].

### 2.8 Statistical Analysis

Descriptive statistics were used to summarize participant demographics and genotype distributions. Continuous variables are presented as mean ± standard deviation (SD), and categorical variables as frequencies and percentages. Differences between ART groups were assessed using unpaired Student's *t*‐tests for continuous variables and chi‐square tests for categorical variables. Associations between SNPs and CVD risk were assessed using genotypic, dominant, recessive, and allelic models for each polymorphism: *ABCB1* (rs1045642), *APOE* (rs429358 and rs7412), and *PNPLA3* (rs738409). Model selection was based on allele frequencies and biological plausibility. Associations between SNPs and D:A:D CVD risk scores were evaluated using logistic regression and generalized linear models (analysis of covariance), adjusting for sex, age, plasma HIV viral load, CD4 count, smoking status, ART regimen, and lipid profiles. Given the exploratory nature of this study targeting specific candidate genes, no post hoc corrections for multiple comparisons were applied. This approach was chosen to avoid Type II errors in identifying potential associations within these relevant biological pathways, though *p*‐values near the significance threshold should be interpreted with caution. Effect sizes were reported as odds ratios (ORs) with 95% confidence intervals (CIs) to convey both magnitude and precision [23]. To ensure data reliability, several quality control measures were applied. All SNPs included in the analysis had a call rate greater than 98%, and duplicate samples were genotyped to assess reproducibility, achieving concordance rates exceeding 99%. Additionally, Hardy–Weinberg equilibrium (HWE) was tested for each SNP within the study population, and variants showing significant deviation from HWE (*p* < 0.001) were excluded from further analysis [29]. These steps ensured high accuracy and consistency in the genotyping process. Genotype distributions were assessed for HWE using chi‐square tests. Allele frequencies were calculated based on observed genotype counts, and 95% CIs were computed using the normal approximation method [29, 30].

*p‐*values were reported with the corresponding test and data compared. Reporting followed standard conventions: *p* < 0.001 for values below 0.001; three decimals for 0.001–0.01; two decimals for ≥ 0.01; and *p* > 0.99 for values above 0.99. Statistical significance was set at *p* < 0.05, but interpretation emphasized effect sizes and CIs rather than *p*‐values alone.

All statistical analyses were conducted using SAS software, version 9.4 (SAS Institute Inc., Cary, NC, USA) on a 64‐bit Windows 11 operating system running on an Intel® Core™ i7 processor with 16 GB RAM.

## 3 Results

### 3.1 Baseline Characteristics of the Participants

Baseline characteristics of 337 participants are summarized in Table 1, with most being male (322/337; 95.6%) and a mean age of 34.6 years (SD 10.2). Nearly half of the participants (157/337; 46.6%) had undetectable plasma viral load, and the mean CD4 count was 476 cells/mm³ (SD 258.08). The average BMI (kg/m²) was 22.6 (SD 4.0), with obesity (BMI > 30 kg/m²) observed in 5.0% (17/337). Metabolic abnormalities were common, particularly low HDL cholesterol (202/337; 59.9%), whereas elevated blood glucose was least frequent (9/337; 2.9%). The prevalence of MetS was 24.0% (81/337).

**Table 1:** Baseline characteristics of the participants (*N* = 337) in this study.

Variables	*N* (%)
Sex (Male)	322/337 (95.6)
Age (mean ± SD, years)	34.63 ± 10.17
PVL (copies/mL)	
PVL < 20	157/337 (46.6)
20 ≤ PVL ≤ 2000	55/337 (16.3)
PVL > 2000	125/337 (37.1)
CD4 count (mean ± SD, cells/μL)	476.21 ± 258.08
Weight (mean ± SD, kg)	66.12 ± 12.39
BMI (mean ± SD, kg/m²)	22.64 ± 4.03
Obesity (BMI > 30 kg/m²)	17/337 (5.0)
SBP (mean ± SD, mmHg)	123.19 ± 15.81
DBP (mean ± SD, mmHg)	77.65 ± 11.15
TG (mean ± SD, mg/dL)	146.36 ± 105.52
> 150	116/337 (34.4)
≤ 150	221/337 (65.6)
TC (mean ± SD, mg/dL)	167.01 ± 39.89
> 200	56/337 (16.6)
≤ 200	281/337 (83.4)
HDL‐C (mean ± SD, mg/dL)	38.34 ± 11.90
> 40	135/337 (40.1)
≤ 40	202/337 (59.9)
LDL‐C (mean ± SD, mg/dL)	98.28 ± 28.39
> 100	145/337 (43.0)
≤ 100	192/337 (57.0)
Blood sugar (Mean ± SD, mg/dL)	100.42 ± 19.17
Blood sugar > 126	10/337 (3.0)
Blood sugar ≤ 126	327/337 (97.0)
Self‐reported DM	9/337 (2.7)
Baseline MetS	81/337 (24.0)
MetS Medication	
Antihypertensive drugs	22/337 (6.5)
Hypoglycemic agents	12/337 (3.6)
Lipid‐lowering agents	51/337 (15.1)
Smoker	123/337 (36.5)
Baseline D:A:D (R) CVD 5‐year risk score (mean ± SD)	1.71 ± 3.18
Baseline risk score ≥ 5	30/337 (8.9)
Baseline risk score < 5	307/337 (91.1)
Baseline D:A:D (R) CVD 10‐year risk score (mean ± SD)	3.37 ± 5.81
Baseline risk score ≥ 5	54/337 (16.0)
Baseline risk score < 5	283/337 (84.0)
Baseline D:A:D (F) CVD 5‐year risk score (mean ± SD)	1.72 ± 3.14
Baseline risk score ≥ 5	32/337 (9.5)
Baseline risk score < 5	305/337 (90.5)
SNPs	
rs1045642	
G/G	135/337 (40.1)
G/A	162/337 (48.1)
A/A	40/337 (11.9)
rs429358	
T/T	271/337 (80.4)
T/C	63/337 (18.7)
C/C	3/337 (0.9)
rs7412	
C/C	293/337 (86.9)
C/T	44/337 (13.1)
T/T	0 (0)
rs738409	
C/C	145/337 (43.0)
C/G	152/337 (45.1)
G/G	40/337 (11.9)

*Note:* Categorical variables are presented as *n* (%). Continuous data are presented as mean ± standard deviation (SD). Differences between groups were assessed using Student's *t*‐test for continuous variables and Chi‐square test (or Fisher's exact test where appropriate) for categorical variables. rs1045642 is a variant within the *ABCB1* gene, rs7412 and rs429358 are *APOE* variants, and rs738409 is a variant of the *PNPLA3* gene.

Abbreviations: CVD, cardiovascular disease; D:A:D (F), full model from the data collection on adverse events of anti‐HIV drugs study; D:A:D (R), risk equation from the data collection on adverse events of anti‐HIV drugs study; DBP, diastolic blood pressure; DM, diabetic mellitus; HDL‐C, high‐density lipoprotein cholesterol; LDL‐C, low‐density lipoprotein cholesterol; MetS, metabolic syndrome; PVL, plasma HIV viral load; SBP, systolic blood pressure; SNP, single nucleotide polymorphism; TC, total cholesterol; TG, triglyceride.

CVD risk scores were generally low: fewer than 10% (32/337) had a 5‐year risk ≥ 5%, while 16%–18% (54–59/337) had a 10‐year risk ≥ 5%. Genotype distributions showed *ABCB1*(rs1045642) G/A as the most frequent (162/337; 48.1%), *APOE*(rs429358) T/T predominance (271/337; 80.4%), *APOE*(rs7412) C/C (293/337; 86.9%), and *PNPLA3*(rs738409) variants distributed across C/C (145/337; 43.0%) and C/G (153/337; 45.1%).

### 3.2 Differences Between Participants in the Antiretroviral Drug Switch and Continue Groups

To examine factors associated with switching ART among PLWH, participants were categorized into two groups based on 5‐year follow‐up medication records: the ART switch group (*n* = 238) and the continuation group (*n* = 99). Table 2 compares switch and continuation groups revealed no significant differences in age, BMI, blood pressure, or lipid profiles (*p* > 0.05). Lipid‐lowering agent use was higher in the switch group (43/238; 18.07% vs. 8/99; 8.08%; OR: 2.51, 95% CI: 1.13–5.55; *p* = 0.02). The switch group had higher D:A:D (R) CVD risk scores for both 5‐year (mean difference: 0.62, 95% CI: 0.03–1.21; *p* = 0.04) and 10‐year estimates (mean difference: 1.13, 95% CI: 0.01–2.25; *p* = 0.05). Similarly, D:A:D (F) CVD risk scores were higher in the switch group for 5‐year (mean difference: 0.67, 95% CI: 0.08–1.26; *p* = 0.03) and 10‐year estimates (mean difference: 1.26, 95% CI: 0.13–2.39; *p* = 0.03).

**Table 2:** Differences on basic characteristics between participants in antiretroviral drug switch group and continue group.

Variables	Antiretroviral drug switch group (*n* = 238)	Continue group (*n* = 99)	Effect size (95% CI)	*p*‐value
Sex (Male)	235/238 (96.71%)	93/99 (93.00%)	OR: 5.05 (1.24, 20.63)	0.13
Age (Mean ± SD, years)	35.15 ± 10.52	33.39 ± 9.21	Diff: 1.76 (−0.49, 4.01)	0.15
PVL (copies/mL)				
< 20	120/238 (49.38%)	40/99 (40.00%)	OR: 1.50 (0.93–2.41)	0.12
20 ≤ PVL ≤ 2000	39/238 (16.05%)	17/99 (17.00%)	OR: 0.95 (0.51–1.77)	> 0.99
PVL > 2000	84/238 (34.57%)	43/99 (43.00%)	OR: 0.71 (0.44–1.15)	0.20
CD4 count (Mean ± SD)	465.70 ± 249.80	503.80 ± 277.90	Diff: −38.10 (−101.38, 25.18)	0.21
Weight (Mean ± SD)	66.62 ± 12.60	64.92 ± 11.85	Diff: 1.70 (−1.13, 4.53)	0.25
BMI (Mean ± SD, cells/μL)	22.71 ± 4.08	22.48 ± 3.91	Diff: 0.23 (−0.70, 1.16)	0.64
Obesity (BMI > 30, kg/m²)	14/238 (5.88%)	3/99 (3.03%)	OR: 2.00 (0.56, 7.12)	0.41
SBP (Mean ± SD, mmHg)	122.80 ± 15.90	124.00 ± 15.63	Diff: −1.20 (−4.88, 2.48)	0.53
DBP (Mean ± SD, mmHg)	78.21 ± 10.80	76.31 ± 11.88	Diff: 1.90 (−0.81, 4.61)	0.15
TG (Mean ± SD, mg/dL)	149.30 ± 110.40	139.30 ± 92.86	Diff: 10.00 (−13.05, 33.05)	0.40
TG > 150	82/238 (34.45%)	34/99 (34.34%)	OR: 1.00 (0.61–1.65)	> 0.99
TG ≤ 150	156/238 (65.55%)	65/99 (65.66%)	OR: 0.9983 (0.8425, 1.1829)	0.98
TC l (Mean ± SD, mg/dL)	169.50 ± 40.57	160.90 ± 37.72	Diff: 8.60 (−0.44, 17.64)	0.07
TC > 200	42/238 (17.65%)	14/99 (14.14%)	OR: 1.30 (0.67–2.51)	0.53
TC ≤ 200	196/238 (82.35%)	85/99 (85.86%)	OR: 0.77 (0.40–1.48)	0.53
HDL cholesterol (Mean ± SD, mg/dL)	38.62 ± 11.59	37.64 ± 12.66	Diff: 0.98 (−1.92, 3.88)	0.49
HDL cholesterol > 40	92/238 (38.66%)	43/99 (43.43%)	OR: 0.82 (0.51–1.32)	0.49
HDL cholesterol ≤ 40	146/238 (61.34%)	56/99 (56.57%)	OR: 1.22 (0.76–1.96)	0.49
LDL cholesterol (Mean ± SD, mg/dL)	99.96 ± 29.03	94.21 ± 26.50	Diff: 5.75 (−0.64, 12.14)	0.09
LDL cholesterol > 100	107/238 (44.96%)	38/99 (38.38%)	OR: 1.31 (0.81–2.12)	0.32
LDL cholesterol ≤ 100	131/238 (55.04%)	61/99 (61.62%)	OR: 0.76 (0.47–1.23)	0.32
Blood sugar (Mean ± SD, mg/dL)	100.20 ± 16.88	100.90 ± 23.88	Diff: −0.70 (−5.87, 4.47)	0.81
Blood sugar > 126	6/238 (2.52%)	4/99 (4.04%)	OR: 0.61 (0.17–2.23)	0.69
Blood sugar ≤ 126	232/238 (97.48%)	95/99 (95.96%)	OR: 1.63 (0.45–5.90)	0.69
Self‐reported DM	7 (2.94%)	2/99 (2.02%)	OR: 1.47 (0.30, 7.20)	0.63
Baseline metabolic syndrome	60/238 (25.21%)	21/99 (21.21%)	OR: 1.25 (0.71, 2.20)	0.43
Anti‐hypertension drugs	18/238 (7.56%)	4/99 (4.04%)	OR: 1.94 (0.64, 5.90)	0.23
Hypoglycemic agents	9/238 (3.78%)	3/99 (3.03%)	OR: 1.26 (0.33, 4.75)	0.73
Lipid‐lowering agents	43/238 (18.07%)	8/99 (8.08%)	OR: 2.51 (1.13, 5.55)	0.02*
Smoker	91/238 (38.24%)	32/99 (32.32%)	OR: 1.30 (0.79, 2.13)	0.30
D:A:D (R) CVD 5‐year risk(Mean ± SD)	1.89 ± 3.55	1.27 ± 1.95	Diff: 0.62 (0.03, 1.21)	0.04*
Baseline risk score ≥ 5 (Set 1)	25/238 (10.50%)	5/99 (5.05%)	OR: 2.21 (0.82–5.94)	0.16
Baseline risk score ≥ 5 (Set 2)	41/238 (17.23%)	13/99 (13.13%)	OR: 1.38 (0.70–2.70)	0.44
D:A:D (R) CVD 10‐year risk (Mean ± SD)	3.70 ± 6.43	2.57 ± 3.86	Diff: 1.13 (0.01, 2.25)	0.05
Baseline risk score ≥ 5	26/238 (10.92%)	6/99 (6.06%)	OR: 1.90 (0.76–4.77)	0.24
Baseline risk score ≥ 5	44/238 (18.49%)	15/99 (15.15%)	OR: 1.27 (0.67–2.41)	0.56
D:A:D (F) CVD 5‐year risk (Mean ± SD)	1.92 ± 3.51	1.25 ± 1.93	Diff: 0.67 (0.08, 1.26)	0.03*
Baseline risk score < 5	213/238 (89.50%)	94/99 (94.95%)	OR: 0.45 (0.17–1.22)	0.16
Baseline risk score < 5	197/238 (82.77%)	86/99 (86.87%)	OR: 0.73 (0.37–1.42)	0.44
D:A:D (F) CVD 10‐year risk (Mean ± SD)	3.81 ± 6.56	2.55 ± 3.84	Diff: 1.26 (0.13, 2.39)	0.03*
Baseline risk score < 5	212/238 (89.08%)	93/99 (93.94%)	OR: 0.53 (0.21–1.32)	0.24
Baseline risk score < 5	194/238 (81.51%)	84/99 (84.85%)	OR: 0.79 (0.42–1.49)	0.56

*Note:* Categorical variables as frequencies and percentages. Continuous data are presented as mean ± standard deviation (SD). Statistical comparisons were performed using Student's *t*‐test for continuous variables and Chi‐square test for categorical variables. Effect sizes are reported as OR with 95%CI for categorical variables and Mean Differences with 95% CI for continuous variables. *p*‐values were reported with the corresponding test and data compared. Reporting followed standard conventions: *p* < 0.001 for values below 0.001; three decimals for 0.001–0.01; two decimals for ≥ 0.01; and *p* > 0.99 for values above 0.99. *A *p*‐value < 0.05 was considered statistically significant.

Abbreviations: CI, confidence interval; CVD, cardiovascular disease; D:A:D (F), full model from the data collection on adverse events of anti‐HIV drugs study; D:A:D (R), risk equation from the data collection on adverse events of anti‐HIV drugs study; DBP, diastolic blood pressure; Diff, mean difference; DM, diabetic mellitus; HDL‐C, high‐density lipoprotein cholesterol; LDL‐C, low‐density lipoprotein cholesterol; MetS, metabolic syndrome; OR, odds ratio; PVL, plasma HIV viral load; SBP, systolic blood pressure; SD, standard deviation; TC, total cholesterol; TG, triglyceride.

### 3.3 Genotype Distributions, HWE Statistics, and Allele Frequencies Among PLWH by Antiretroviral Drug Switch Status

Genotype distributions and their association with ART switching are summarized in Table 3. No statistically significant differences were observed between the ART switch and continuation groups (*p* > 0.05). For *ABCB1*(rs1045642), the A/A genotype was more frequent in the switch group (33/238; 13.9%) than in the continuation group (7/99; 7.1%) (OR: 2.12; 95% CI: 0.90–4.96; *p* = 0.12). Similarly, *PNPLA3*(rs738409) G/G genotype appeared slightly higher in the switch group (30/238; 12.6%) compared to the continuation group (10/99;10.1%), but this difference was not significant (OR: 1.28; 95% CI: 0.60–2.74; *p* = 0.64). Risk differences and ratios for all SNPs were close to null, indicating minimal effect sizes.

**Table 3:** Differences on polymorphism genotype distributions between participants in antiretroviral drug switch group and continue group.

SNPs	Genotype	ART switch group (*n* = 238) %	Continue group (*n* = 99) %	Risk Diff (95% CI)	Risk ratio (95% CI)	OR (95% CI)	*p*‐value
rs1045642	G/G	94/238 (39.5%)	41/99 (41.41%)	−0.0192 (−0.1344 to 0.0960)	0.9537 (0.7192–1.2646)	0.92 (0.57–1.49)	0.84
	G/A	111/238 (46.64%)	51/99 (51.52%)	−0.0488 (−0.1658 to 0.0683)	0.9053 (0.7161–1.1446)	0.82 (0.51–1.32)	0.49
	A/A	33/238 (13.87%)	7/99 (7.07%)	0.0679 (0.0010–0.1349)	1.9610 (0.8979–4.2830)	2.12 (0.90–4.96)	0.12
rs429358	T/T	190/238 (79.83%)	81/99 (81.82%)	−0.0199 (−0.1114 to 0.0716)	0.9757 (0.8717–1.0921)	0.88 (0.48–1.60)	0.79
	T/C	46/238 (19.33%)	17/99 (17.17%)	0.0216 (−0.0681 to 0.1112)	1.1256 (0.6796–1.8641)	1.16 (0.63–2.13)	0.76
	C/C	2/238 (0.84%)	1/99 (1.01%)	−0.0017 (−0.0246 to 0.0212)	0.8319 (0.0763–9.0705)	0.83 (0.07–9.27)	> 0.99
rs7412	C/C	204/238 (85.71%)	89/99 (89.9%)	−0.0418 (−0.1160 to 0.0323)	0.9535 (0.8767–1.0370)	0.67 (0.32–1.42)	0.39
	C/T	34/238 (14.29%)	10/99 (10.1%)	0.0418 (−0.0323 to 0.1160)	1.4143 (0.7273–2.7500)	1.48 (0.70–3.13)	0.39
rs738409	C/C	98/238 (41.18%)	47/99 (47.47%)	−0.0630 (−0.1795 to 0.0536)	0.8673 (0.6708–1.1214)	0.77 (0.48–1.24)	0.35
	C/G	110/238 (46.22%)	42/99 (42.42%)	0.0379 (−0.0782 to 0.1541)	1.0894 (0.8339–1.4233)	1.17 (0.73–1.87)	0.60
	G/G	30/238 (12.61%)	10/99 (10.1%)	0.0250 (−0.0478 to 0.0979)	1.2479 (0.6346–2.4539)	1.28 (0.60–2.74)	0.64

*Note:* Categorical variables as frequencies and percentages. Continuous data are presented as mean ± standard deviation (SD). Genotype distribution differences between groups were analyzed using the Chi‐square test. OR and Risk Ratios with 95% CI were calculated to assess the strength of associations. ORs and *p*‐values were calculated using an allelic model (comparing major vs. minor alleles) and genotypic model comparisons as specified. *p*‐values were reported with the corresponding test and data compared. Reporting followed standard conventions: *p* < 0.001 for values below 0.001; three decimal places for values between 0.001 and 0.01; two decimal places for values ≥ 0.01; and *p* > 0.99 for values above 0.99. *A *p*‐value < 0.05 was considered statistically significant. rs1045642 is a variant within the *ABCB1* gene, rs7412 and rs429358 are *APOE* variants, and, rs738409 is a variant of the *PNPLA3* gene.

Abbreviations: ART, antiretroviral therapy; CI, confidence interval; Diff, mean difference; OR, odds ratio; SNP, single nucleotide polymorphism.

Genotype distributions and allele frequencies for the four candidate SNPs, assessed for HWE, are presented in Tables 4 and 5. Genotype distributions for *ABCB1* (rs1045642) were G/G:;40.1% (135/337), G/A: 48.1% (162/337), A/A: 11.8% (40/337), allele frequencies: G: 0.628 (95% CI: 0.585–0.672), A: 0.372 (95% CI: 0.328–0.415). For *APOE*(rs429358), T/T: 80.4% (2271/337), T/C: 18.7% (63/337), C/C: 0.9% (3/337); allele frequencies: T: 0.895, C: 0.105. *APOE*(rs7412) showed C/C: 86.9%(293/337), C/T: 13.1% (44/337); allele frequencies: C: 0.929, T: 0.071. *PNPLA3*(rs738409) frequencies were C/C: 43.0% (145/337), C/G: 45.1% (152/337), G/G: 11.9% (40/337); allele frequencies: C: 0.643, G: 0.357. All SNPs were consistent with HWE (*p* > 0.05), indicating no significant deviation.

**Table 4:** Genotype distribution and Hardy–Weinberg equilibrium analysis for four candidate SNPs among study participants (*N* = 337).

SNPs	Genotype	*N*	%	Observed	Expected	Chi‐square	95% CI	*p*‐value
rs1045642	G/G	135/337	40.06	135	138.45	0.34	120.74, 156.15	0.85
	G/A	162/337	48.07	162	155.11	0.34	137.18, 173.04	0.85
	A/A	40/337	11.87	40	43.45	0.34	31.39, 55.50	0.85
rs429358	T/T	271/337	80.42	271	271.53	0.05	257.30, 285.77	0.97
	T/C	63/337	18.69	63	61.94	0.05	48.00, 75.87	0.97
	C/C	3/337	0.89	3	3.53	0.05	0.00, 7.20	0.97
rs7412	C/C	293/337	86.94	293	294.44	1.54	282.48, 306.39	0.46
	C/T	44/337	13.06	44	41.13	1.54	29.35, 52.91	0.46
	T/T	0/337	0.00	0	1.44	1.54	0.00, 3.78	0.46
rs738409	C/C	145/337	43.03	145	144.93	0.00	127.12, 162.74	> 0.99
	C/G	152/337	45.10	152	152.14	0.00	134.24, 170.05	> 0.99
	G/G	40/337	11.87	40	39.93	0.00	28.30, 51.56	> 0.99

*Note:* Genotype distributions were calculated from observed data. HWE was assessed using the chi‐square goodness‐of‐fit test. A *p*‐value greater than 0.05 indicates no significant deviation from HWE. Values are presented as *n* (%) and observed versus expected counts. *p*‐values were reported with the corresponding test and data compared. Reporting followed standard conventions: *p* < 0.001 for values below 0.001; three decimal places for values between 0.001 and 0.01; two decimal places for values ≥ 0.01; and *p* > 0.99 for values above 0.99. rs1045642 is a variant within the *ABCB1* gene, rs7412 and rs429358 are *APOE* variants, and, rs738409 is a variant of the *PNPLA3* gene.

Abbreviations: CI, confidence interval; SNPs, single nucleotide polymorphisms.

**Table 5:** Allele frequencies and Hardy–Weinberg equilibrium test for four candidate SNPs. (*N* = 337).

SNPs	Allele	*n*	Frequency	95% CI	*p*‐value
rs1045642	G	216/337	0.6409	0.6047, 0.6772	0.85
	A	121/337	0.3591	0.3228, 0.3953	0.85
rs429358	T	302.5/337	0.8976	0.8747, 0.9205	0.97
	C	34.5/337	0.1024	0.0795, 0.1253	0.97
rs7412	C	315/337	0.9347	0.9161, 0.9534	0.46
	T	22/337	0.0653	0.0466, 0.0839	0.46
rs738409	C	221/337	0.6558	0.6199, 0.6917	> 0.99
	G	116/337	0.3442	0.3083, 0.3801	> 0.99

*Note:* Allele frequencies were calculated based on observed genotypes. Allele frequencies are expressed as proportions with 95% CI. HWE was assessed using the chi‐square test. Reporting followed standard conventions: *p* < 0.001 for values below 0.001; three decimal places for values between 0.001 and 0.01; two decimal places for values ≥ 0.01; and *p* > 0.99 for values above 0.99. A *p*‐value greater than 0.05 indicates no significant deviation from HWE. rs1045642 is a variant within the *ABCB1* gene, rs7412 and rs429358 are *APOE* variants, and, rs738409 is a variant of the *PNPLA3* gene.

Abbreviations: CI, confidence interval; SNPs, single nucleotide polymorphisms.

### 3.4 Association Between SNPs in the Lipoprotein Gene and the Risk of CVD

Differences in D:A:D CVD risk scores across genotypes for the four SNPs are presented in Table 6. Statistical analyses included genotypic, dominant, recessive, and allelic models for each SNP. For example, the association between *PNPLA3* (rs738409) and CVD risk was evaluated under all four models, with significant findings observed primarily in the genotypic models. Similar approaches were applied to *ABCB1* (rs1045642) and *APOE* (rs429358, rs7412). For *ABCB1*(rs1045642), mean 5‐ and 10‐year risk scores did not differ significantly among genotypes (*p*‐values ranged from *p* = 0.39 to *p* = 0.51). For *APOE*(rs429358), no significant associations were observed across genotypes for any risk score (*p*‐values ranged from *p* = 0.41 to *p* = 0.61). For *APOE*(rs7412), carriers of the T allele (C/T genotype) exhibited higher mean risk scores compared to C/C, but differences were not statistically significant (*p*‐values ranged from *p* = 0.07 to *p* = 0.14). For PNPLA3(rs738409), significant associations were detected for the D:A:D (R) 10‐year risk score (*p* = 0.03) and marginal associations for the D:A:D (R) 5‐year (*p* = 0.05) and D:A:D (F) 10‐year risk scores (*p* = 0.05). The C/C genotype showed the highest mean risk scores, while G/G had the lowest. Overall, *PNPLA3*(rs738409) demonstrated the strongest evidence of association with increased CVD risk, particularly for long‐term risk estimates, whereas other SNPs showed no significant differences.

**Table 6:** Association between SNPs and D:A:D cardiovascular disease risk scores among study participants (*N* = 337).

SNPs	Genotype	*n* (%)	D:A:D (R) 5‐year (95% CI)	D:A:D (R) 10‐year (95% CI)	D:A:D (F) 5‐year (95% CI)	D:A:D (F) 10‐year (95% CI)
rs1045642	G/G	135/337 (40.06)	1.64 (0.95–2.33)	3.19 (1.98–4.40)	1.56 (0.95–2.17)	3.06 (1.95–4.17)
	G/A	162/337 (48.07)	1.61 (1.22–2.00)	3.23 (2.46–4.00)	1.36 (1.05–1.67)	2.75 (2.14–3.36)
	A/A	40/337 (11.87)	0.98 (0.59–1.37)	2.00 (1.21–2.79)	1.03 (0.54–1.52)	2.10 (1.12–3.08)
*p*‐value			0.45	0.39	0.51	0.50
rs429358	T/T	271/337 (80.42)	1.50 (1.10–1.90)	2.96 (2.25–3.67)	1.41 (1.06–1.76)	2.80(2.15–3.45)
	T/C	63/337 (18.69)	1.80 (1.11–2.49)	3.60 (2.25–4.95)	1.46 (0.94–1.98)	2.97 (1.94–4.00)
	C/C	3/337 (0.89)	0.32 (0.12–0.52)	0.65 (0.24–1.06)	0.27 (0.11–0.43)	0.56 (0.23–0.89)
*p*‐value			0.53	0.41	0.61	0.53
rs7412	C/C	293/337 (86.94)	1.40 (1.15–1.65)	2.84 (2.35–3.33)	1.30 (1.08–1.52)	2.64 (2.20–3.08)
	C/T	44/337 (13.06)	2.32 (0.37–4.27)	4.29 (0.96–7.62)	2.00 (0.33–3.67)	3.76 (0.83–6.69)
*p*‐value			0.07	0.09	0.10	0.14
rs738409	C/C	145/337 (43.03)	1.97 (1.25–2.69)	3.83 (2.54–5.12)	1.71 (1.10–2.32)	3.37 (2.25–4.49)
	C/G	152/337 (45.10)	1.31 (1.05–1.57)	2.67 (2.14–3.20)	1.28 (1.01–1.55)	2.60 (2.06–3.14)
	G/G	40/337 (11.87)	0.75 (0.56–0.94)	1.54 (1.16–1.92)	0.67 (0.49–0.85)	1.39 (1.03–1.75)
*p*‐value			0.05	0.03*	0.07	0.05

*Note:* CI: confidence interval; D:A:D (R) = Risk equation from the Data Collection on Adverse Events of Anti‐HIV Drugs study; D:A:D (F) = Full model from the Data Collection on Adverse Events of Anti‐HIV Drugs study; SNP: single nucleotide polymorphism. Statistical comparisons across genotypes within each SNP group were performed using analysis of covariance (ANCOVA; generalized linear models), with *p*‐values representing differences adjusted for sex, age, plasma viral load, CD4 count, and smoking status. Values are presented as risk estimates with 95%CI. Reporting followed standard conventions: *p* < 0.001 for values below 0.001; three decimal places for values between 0.001 and 0.01; two decimal places for values ≥ 0.01; and *p* > 0.99 for values above 0.99.

* A *p*‐value < 0.05 was considered statistically significant. rs1045642 is a variant within the *ABCB1* gene, rs7412 and rs429358 are *APOE* variants, and rs738409 is a variant of the *PNPLA3* gene.

## 4 Discussion

The findings of this study suggest that maintaining a consistent ART regimen is associated with a reduction in CVD risk scores among HIV patients undergoing treatment. In contrast, patients who switch ART regimens tend to exhibit an increase in CVD risk scores and a higher likelihood of developing CVD. While various mechanisms may contribute to the elevated CVD risk associated with ART modification, this study highlights the potential involvement of genetic polymorphisms rs738409 (a variant of the *PNPLA3* gene) and rs7412 (a variant of the *APOE* gene) [16, 18, 19, 22]. Specifically, the presence of the C allele of rs738409 (*PNPLA3*) and the T allele of *APOE*(rs7412) appears to be associated with an increased susceptibility to CVD in PLWH in this study, potentially influencing their decision—whether active or passive—to alter their ART regimen during treatment. These variants may affect lipid metabolism and inflammation, key drivers of CVD risk. Associations with *p*‐values near 0.05 should be viewed as trends, not definitive evidence. While literature supports a possible link, confirmation requires larger, well‐designed studies.

The observed associations between rs738409 (*PNPLA3*) and rs7412 (*APOE*) and CVD risk in PLWH may be explained by mechanisms primarily involving lipid metabolism and inflammation. The C allele of rs738409(*PNPLA3*) was associated with higher CVD risk scores in our study, differing from reports in the general population where the G allele is typically linked to hepatic steatosis and metabolic risk. This discrepancy suggests that allelic effects may be context‐dependent, varying with disease status, outcome definitions (risk scores vs. clinical events), and population characteristics. In PLWH, the interplay between HIV infection, chronic inflammation, and ART may modulate the functional impact of this variant, distinct from mechanisms observed in uninfected cohorts [22, 31, 32]. These genetic variants may affect the expression or activity of genes involved in the development or progression of CVD, such as those related to lipid metabolism, inflammation, and oxidative stress [22, 33, 34]. Similarly, the T allele of rs7412 (*APOE*) influences lipid transport and clearance, contributing to elevated LDL and total cholesterol levels [33, 34]. Similarly, the T allele of rs7412 (*APOE)* influences lipid transport and clearance, contributing to elevated LDL and total cholesterol levels [33, 34]. These effects may be amplified by ART, as certain regimens are known to increase TG and LDL cholesterol [35, 36, 37, 38, 39]. Chronic inflammation and immune activation induced by HIV infection further interact with these genetic predispositions, promoting atherogenesis [39]. The higher CVD risk scores may be more likely to adopt unhealthy behaviors in PLWH, such as smoking, poor diet, or sedentary lifestyle, which can further exacerbate their CVD risk [40, 41, 42]. These pathways—lipid dysregulation and inflammation—represent the most plausible biological routes supported by current evidence and our findings, rather than behavioral factors, which were not directly assessed in this study.

All SNPs met HWE, indicating no genotyping errors or population stratification. Reporting allele frequencies with 95% CI provides a clearer view of genetic variation than *p*‐values alone. Genetic variation may influence CVD risk in PLWH on ART, suggesting potential use of genetic screening for personalized risk stratification. Given modest effect sizes and overlapping CIs, SNP‐based prediction should complement—not replace—traditional tools [31, 35]. Our predominantly male, single‐ethnicity sample limits generalizability; future studies should include diverse cohorts. Clinically, genetic data could guide closer monitoring and ART choices, offering a step toward precision HIV care [32, 33, 34].

This study evaluated whether genetic polymorphisms in lipid‐related genes influence ART switching among PLWH in Taiwan. No significant associations were found between the four candidate SNPs and ART modification over 5 years. Although the *ABCB1* variant rs1045642 A/A genotype showed a trend toward higher switching, the effect was not statistically significant. These findings suggest that ART switching is primarily driven by clinical factors such as drug toxicity, resistance, and comorbidities rather than host genetic variation, consistent with previous reports on the limited impact of SNPs on ART‐related dyslipidemia and treatment outcomes [31, 35]. Moreover, while variants such as rs738409 (*PNPLA3*) have been linked to liver disease progression in HIV/HCV coinfection [32] and *APOE* polymorphisms to CVD risk [33], our data did not show significant associations with ART switching. Larger studies and functional analyses are needed to clarify potential genetic contributions to ART tolerability and metabolic outcomes [34]. Compared to similar studies in the past, this study has a large sample size and sufficient statistical power for analysis [25, 26]. In addition, the genetic and biochemical data collected in this study are complete, providing a more comprehensive evaluation. More importantly, this study has a long follow‐up time, allowing for well‐defined ART switch and continue groups throughout long‐term observation and for calculating D:A:D 5‐ and 10‐year risk scores.

However, several limitations should be acknowledged. First, the study sample is predominantly male (95.6%), which may restrict the generalizability of findings to female individuals living with HIV. This gender imbalance reflects the national epidemiological trend in Taiwan, where, as of December 2023, 42,019 (94.93%) of reported HIV cases were male and only 2244 (5.07%) were female [43]. Nonetheless, this demographic skew has been acknowledged as a study limitation. Second, as the majority of the sample belongs to the Han ethnicity, additional validation is required to assess the applicability of these results to other ethnic groups. Third, the study did not include an HIV‐negative control group, which limits the ability to determine whether the observed genetic associations are specific to PLWH or also present in the general population. Future studies incorporating HIV‐negative controls would help clarify the broader relevance of these findings. Fourth, this study examined multiple SNPs and outcomes, no correction for multiple comparisons was applied, increasing the risk of false positives. Additionally, while single‐locus associations were analyzed, haplotype analysis was not performed, which could have provided further insight into the combined effects of genetic variants. Findings should be interpreted cautiously, and future studies should use haplotype‐based approaches and apply appropriate corrections and validate results in independent cohorts. Finally, we acknowledge the lack of formal power analysis as a limitation and recommend that future studies include power calculations to ensure adequate statistical power.

However, given genetic and environmental heterogeneity across populations, replication studies in larger and more ethnically diverse cohorts are essential to confirm associations and assess generalizability. Future research should examine gene–environment interactions, longitudinal outcomes, and the integration of genetic risk scores into HIV care. Clinically, identifying *PNPLA3* or *APOE* risk alleles could guide personalized interventions, including intensified CVD monitoring, tailored lifestyle counseling, and early pharmacologic strategies. Incorporating genetic markers into HIV care pathways may advance precision medicine and improve long‐term CVD outcomes. Evidence of variability in *PNPLA3* and *APOE* allele frequencies and their effects on lipid metabolism and liver disease among PLWH highlights population‐specific factors influencing CVD risk [44, 45].

## 5 Conclusion

Polymorphisms in *PNPLA3* and *APOE* genes are associated with higher CVD risk among PLWH in Taiwan. These findings emphasize the role of genetic variations in shaping disease susceptibility and support integrating genetic assessments into clinical decision‐making. Evaluating *PNPLA3* and *APOE* genotypes may help clinicians optimize ART regimens, improving patient outcome, and contributing to more personalized approaches in CVD risk management.

## Author Contributions

**Chia‐Hui Yu:** data curation, investigation, supervision. **Gwo‐Tarng Sheu:** formal analysis. **Chien‐Feng Li:** formal analysis. **Win‐Long Lu:** investigation. **Hao‐Jan Yang:** data curation. **Hung‐Chang Hung:** data curation, supervision. **Yuan‐Ti Lee:** conceptualization, funding acquisition, writing – original draft, writing – review and editing.

## Ethical Statement

The protocol for this study was approved by the Institutional Review Board of Chung Shan Medical University Hospital (IRB No: CS15074). Written informed consent was obtained from all participants prior to their inclusion in the study. Participant confidentiality and anonymity were strictly maintained throughout the research process.

## Conflicts of Interest

The authors declare no conflicts of interest.

## Transparency Statement

The lead author Hung‐Chang Hung and Yuan‐Ti Lee affirms that this manuscript is an honest, accurate, and transparent account of the study being reported; that no important aspects of the study have been omitted; and that any discrepancies from the study as planned (and, if relevant, registered) have been explained.

## Data Availability

Upon reasonable request, the corresponding author, Yuan‐Ti Lee, would provide the data that support the study's conclusions.
